# How to achieve high-quality participation in voluntary environmental regulation: Influencing factors and decision mechanism

**DOI:** 10.1371/journal.pone.0332806

**Published:** 2025-09-23

**Authors:** Feng Pan, Yanni Xu, Lin Wang

**Affiliations:** 1 School of Public Administration and Humanities, Dalian Maritime University, Dalian, LiaoNing, China; 2 School of Economics and Management (Tourism), Dalian University, Dalian, LiaoNing, China; Federal University Otuoke, NIGERIA

## Abstract

This paper aims to explore the influencing factors and their decision mechanism of high-quality participation in voluntary environmental regulation, so as to present an ideal situation of synergistic and effective promotion of voluntary environmental governance. For this purpose, a four-party evolutionary game model of “local governments, enterprises, certification institutions and consumers” is constructed under the framework of voluntary environmental regulation, the interaction laws and equilibrium strategies of the multi-subject behaviors are investigated, then numerical simulation and sensitivity analyses are carried out with the help of MATLAB 2023(a). It is found that: active promotion by local governments, legal certification by certification institutions and strong concern from consumers can all promote high-quality participation in voluntary environmental regulation. Among them, legal certification by certification institutions is the key factor, and consumers’ strong concern has the weakest effect. The environmental governance direction of “local government supervise certification institutions, certification institutions review enterprises” can be established by promoting legal certification. Subsidies increased by local governments will help enterprises choose the qualified application strategy more quickly; penalties by local governments and exposure by the media can help certification institutions choose the legal certification strategy faster. This paper will enrich the theoretical research of voluntary environmental regulation from the perspective of participation effectiveness, supplement the practical basis for the high-quality implementation of voluntary environmental regulation, create a new pattern of multiple co-governance, and consolidate the ecological foundation for high-quality development.

## 1 Introduction

China’s economic and social development has entered the high-quality development stage of accelerating green and low-carbon development, but the structural, root and trend pressures on ecological environmental protection have not yet been fundamentally alleviated. The solution of these problems can not be separated from the use of environmental regulation tools, but also from the efforts of local environmental governance. Voluntary Environmental Regulation is a “third type” of environmental governance tool that lies between command-and-control regulation and market incentive regulation. It is characterized by a non-compulsory nature, incentive mechanisms, and selectivity. Usually initiated by governments, industry associations, or independent third parties, it encourages enterprises to take higher-level environmental protection measures beyond legal standards through forms such as environmental protection certification, eco-labels, and voluntary agreements. The core objective of voluntary environmental regulation is to limit enterprise emissions, stimulate green technological innovation, and ultimately enhance overall environmental performance and promote high-quality economic development. Against the backdrop of the transformation of the global environmental governance system, voluntary environmental regulation, with its flexibility, low administrative costs, and market-driven characteristics, is becoming an important mechanism to address the “rigidity of environmental regulation” and enhance the intrinsic motivation of enterprises for environmental protection. In the context of China’s “ecological civilization construction” and “dual carbon” goals, voluntary environmental regulation is regarded as an important tool to complement traditional mandatory supervision. Its rise is mainly attributed to the following background factors: (1) Regulatory cost constraints: The implementation cost of comprehensive mandatory supervision by the government is high, especially in developing regions with limited law enforcement resources. Voluntary environmental regulation can reduce administrative burdens. (2) Increased market and social pressure: The rise in consumers’ environmental awareness and the development of green finance prompt enterprises to gain competitive advantages through environmental protection actions. (3) Diversification of government performance assessment: Local governments, under the pressure of environmental performance, tend to adopt low-cost and demonstrable voluntary environmental regulations (such as green certification) to showcase governance achievements. (4) International environmental agreements and supply chain pressure: The increasingly strict requirements of multinational companies and international markets for environmental compliance force enterprises to voluntarily meet standards to maintain their positions in the supply chain.

There are essential differences in institutional design between voluntary and non-voluntary environmental regulations. Voluntary environmental regulations are based on market-driven forces and grant enterprises the right to participate voluntarily. Their implementation subjects are diversified, including enterprises, industry associations, and third-party institutions, which guide environmental improvement through reputation mechanisms and policy incentives. In contrast, non-voluntary environmental regulations rely on government coercion, have a single implementation subject, and ensure compliance through legal constraints and administrative penalties. In terms of operational mechanisms, voluntary environmental regulations allow enterprises to flexibly adjust environmental protection investments based on cost-benefit analysis, while non-voluntary environmental regulations require enterprises to bear rigid compliance costs. In terms of governance effects, voluntary environmental regulations are more flexible but have the risk of free-riding, while non-voluntary environmental regulations are more stable but may inhibit innovation. Under the two institutional frameworks of voluntary and non-voluntary environmental regulations, the strategic choices of local governments, enterprises, certification institutions, and consumers show significant differences. For local governments, voluntary environmental regulations provide higher strategic autonomy. On the one hand, there are proactive behaviors such as actively promoting green certifications, creating ecological parks, and attracting investment through environmental labels. On the other hand, they may also show a passive attitude or even tacitly allow false certifications due to the lack of hard assessment pressure and resource constraints. In contrast, local governments under non-voluntary environmental regulations are more focused on fulfilling the hard environmental indicators set by higher authorities, and their behaviors tend to be passive and the degree of strategic differentiation is significantly reduced.There are essential differences in institutional design between voluntary and non-voluntary environmental regulations. Voluntary environmental regulations are based on market-driven forces and grant enterprises the right to participate voluntarily. Their implementation subjects are diversified, including enterprises, industry associations, and third-party institutions, which guide environmental improvement through reputation mechanisms and policy incentives. In contrast, non-voluntary environmental regulations rely on government coercion, have a single implementation subject, and ensure compliance through legal constraints and administrative penalties. In terms of operational mechanisms, voluntary environmental regulations allow enterprises to flexibly adjust environmental protection investments based on cost-benefit analysis, while non-voluntary environmental regulations require enterprises to bear rigid compliance costs. In terms of governance effects, voluntary environmental regulations are more flexible but have the risk of free-riding, while non-voluntary environmental regulations are more stable but may inhibit innovation. Under the two institutional frameworks of voluntary and non-voluntary environmental regulations, the strategic choices of local governments, enterprises, certification institutions, and consumers show significant differences. For local governments, voluntary environmental regulations provide higher strategic autonomy. On the one hand, there are proactive behaviors such as actively promoting green certifications, creating ecological parks, and attracting investment through environmental labels. On the other hand, they may also show a passive attitude or even tacitly allow false certifications due to the lack of hard assessment pressure and resource constraints. In contrast, local governments under non-voluntary environmental regulations are more focused on fulfilling the hard environmental indicators set by higher authorities, and their behaviors tend to be passive and the degree of strategic differentiation is significantly reduced. In the context of voluntary environmental regulations, enterprises also exhibit a dualistic tendency. Some enterprises with the necessary conditions view certification as a strategic tool for market competition, aiming to gain excess profits by enhancing their brand image and accessing green finance. However, others may be driven by opportunistic motives and even seek fraudulent certification to take advantage of policy incentives for short-term gains. In contrast, under non-voluntary environmental regulations, enterprises generally adopt a passive compliance strategy, striving to meet standards at the lowest cost, lacking the economic incentives to exceed compliance requirements. Certification institutions in voluntary environmental regulations coexist with compliance-oriented institutions that strictly adhere to standards based on reputation, and profit-driven institutions that lower the threshold for audits under weak supervision or local tolerance. In non-voluntary environmental regulations, certification is usually designated or strictly authorized by the government, with highly unified standards and procedures, leaving little room for non-compliance. Consumers in voluntary environmental regulations can directly influence enterprises’ environmental strategies through purchasing decisions, with high environmental preference groups providing positive market incentives, while low environmental preference groups limit the market’s constraining effect. In non-voluntary environmental regulations, consumers are more passive in accepting the results of policy constraints, and the guiding effect on enterprise behavior is significantly weakened. Overall, under voluntary environmental regulations, the four types of subjects have higher strategic freedom and more differentiated behaviors, with the core driving mechanism being market incentives and reputation effects. Non-voluntary environmental regulations, on the other hand, rely on legal and administrative pressure to form institutionalized consistency. This difference reveals the potential of voluntary regulations in stimulating the initiative of multiple subjects, as well as the challenges in quality control and institutional constraints of participation.

At present, the theory and practice of voluntary environmental regulation in China are still in their infancy. Among them, the most widely used tool is the ISO 14001 environmental management system. According to statistics from the State Administration for Market Regulation, as of July 2025, a total of 557,291 organizations across the country have obtained ISO 14001 certification, ranking among the top in the world. However, from the overall operation perspective, voluntary environmental regulation still faces multiple challenges during implementation: first, the overall certification rate of enterprises is relatively low, making it difficult to fully leverage the advantages of voluntary environmental regulation; second, the certification market order is chaotic, and false certification is rampant; third, the cost of establishing an environmental management system is high and the cycle is long, while some local governments lack sufficient push and financial and policy support is relatively insufficient. These practical problems reflect that the participation in voluntary environmental regulation in China is still not high. In voluntary environmental regulation, “high-quality participation” refers to the active and effective behavior demonstrated by all stakeholders, including the local governments, enterprises, certification institutions, and consumers. Its core connotations include: local governments actively promoting and guiding the implementation of relevant policies; enterprises applying for certification in accordance with the law and regulations on the basis of having the necessary conditions and continuously improving their environmental performance; certification institutions strictly adhering to standards and procedures to ensure the fairness and authority of certification; and consumers paying close attention and forming positive incentives through market choices. High-quality participation in voluntary environmental regulation is a systematic process involving the collaboration of multiple parties, including local governments, enterprises, certification institutions, and consumers. However, existing research mostly focuses on the compliance and sustainability of enterprises and rarely reveals the interaction effects among multiple subjects and their impact on the effectiveness of regulation from an overall perspective. In view of this, this paper will explore the factors influencing high-quality participation in voluntary environmental regulation from four dimensions: local governments, enterprises, certification institutions, and consumers, to provide theoretical support for achieving multi-party collaborative governance and continuous improvement of environmental performance. Under the context of decentralization in China, local governments enjoy a certain degree of autonomy in the implementation of environmental policies and the allocation of governance resources [1]. The central government supervises the actions of local governments through the Central Environmental Protection Inspection Institution and incorporates environmental performance into the promotion assessment system for local officials to improve and enhance local environmental governance [2]. In recent years, an increasing number of local governments, based on environmental performance considerations, have adopted policy tools such as subsidies and tax incentives to actively encourage enterprises to participate in voluntary environmental regulations. Notably, due to the revenue characteristics of voluntary environmental regulations, their impact is not limited to the participating enterprises themselves but also has spillover effects on non-participating enterprises. On the one hand, non-participating enterprises may be driven by potential participation benefits to proactively increase environmental protection investments to meet participation conditions. On the other hand, participating enterprises, leveraging the reputation and market competitiveness gained from participation, exert stronger competitive pressure on non-participating enterprises, thereby compelling the latter to adopt more proactive environmental strategies to maintain their market position. Additionally, non-participating enterprises may also enhance their environmental governance capabilities and participation willingness by imitating or learning from the advanced environmental management experiences and technologies of participating enterprises [3].

The participation of enterprises is not only restricted by their internal capabilities and management factors, but also influenced by multiple external pressures. The internal drivers of enterprise participation include the environmental awareness and orientation of the management, the learning ability of employees, organizational capabilities, and the experience and traditions within the organization. Yang, based on institutional theory, indicates that internal attractive drivers such as management awareness and leadership, learning ability, and traditional experience have a positive impact on voluntary environmental regulations [4]. Compared with small enterprises, large enterprises are more likely to voluntarily bear the higher certification and maintenance costs for ISO14001 certification [5]. The theory of organizational capabilities further points out that dynamic capabilities (such as knowledge absorption and technological innovation) are the key for enterprises to achieve green transformation through voluntary environmental regulations [6]. The external drivers of enterprise participation include regulatory pressure, social pressure, and market pull. The regulatory pressure from the government actively promotes high-quality participation of enterprises in voluntary environmental regulations [7]. To avoid stricter laws and regulations, enterprises voluntarily invest high costs to improve their environmental performance [8], and government subsidies help enterprises alleviate the financial pressure brought by environmental improvement. Regarding social pressure, the higher the media attention to an enterprise, the higher its environmental performance level [9]. Zhang et al. found that the total number of media reports, the number of follow-up rectification reports, and the number of unrectified reports are significantly positively correlated with the number of times related enterprises are regulated by the government due to environmental issues [10]. In terms of market pull, enterprises that participate in certification and obtain a “green label” can attract more consumers who prefer green products and gain a competitive advantage. To increase market share, enterprises with good environmental performance are more likely to expand into international markets [11], promoting the “quantitative and qualitative improvement” of exports [12] and enhancing their internationalization level [13].

Although green consumption has been frequently mentioned in academic circles and policy practices, systematic research on consumer preferences, behavioral cognition, and their feedback mechanisms to corporate environmental decisions is still insufficient at present. Existing literature indicates that environmental labels can effectively influence consumers’ purchasing choices [14], and green brand images can also help enhance brand loyalty [15]. However, the role of consumers’ willingness to pay, information acquisition channels and trust mechanisms in promoting high-quality participation in voluntary environmental regulations has yet to be structurally explored. In fact, consumers are the most direct perceivers of corporate environmental performance and can provide timely and effective external supervision to enterprises during product use and consumption. At the same time, consumers’ purchasing decisions directly affect the market returns of enterprises, thereby significantly influencing their business strategies and environmental protection investments, and prompting enterprises to more actively respond to the requirements of voluntary environmental regulations under market competition and reputation pressure. Therefore, the behavioral patterns of consumers and their action chains are important links in driving high-quality participation in voluntary environmental regulations. From the perspective of theoretical evolution, research on consumers and their behavioral patterns has shifted from the Theory of Planned Behavior to the Consumption Value Theory. Early studies, based on the Theory of Planned Behavior, emphasized the influence of attitude, subjective norms, and perceived behavioral control on green purchase intentions and actual purchasing behavior. For instance, Zeng Qianqian et al. empirically confirmed that environmental cognition has a significant role in promoting green consumption intentions [16]; Du Jianguo’s research verified that environmental responsibility positively drives green purchasing behavior [17]. Recent studies, however, have focused more on the Consumption Value Theory, concentrating on how information such as eco-labels and green certifications can guide and enhance green consumption by reshaping the functional, social, and emotional values of products. For example, Zhang Xuemu’s empirical analysis demonstrated that eco-labels and the environmental information they convey can effectively enhance consumers’ perception of the green value of products, which in turn translates into green purchasing behavior [18]. In voluntary environmental regulations, this value perception process, combined with consumers’ external supervision function, not only exerts continuous pressure on enterprises for environmental improvement but may also promote the overall improvement of the quality of institutional operation through market feedback mechanisms.

From the perspective of evolutionary game theory, existing research mostly focuses on the interaction between the government and enterprises, while studies on certification institutions as independent game players are still insufficient. In fact, certification institutions have a dual role in voluntary environmental regulation: on the one hand, as third-party auditors, they play the role of “signal mediators”, reducing information asymmetry between the government and enterprises through professional assessment; on the other hand, as market suppliers of certification services, their impartiality directly determines the effectiveness of voluntary environmental regulation. However, in reality, certification institutions often show significant opportunistic tendencies: to maximize profits, they may relax audit standards, make on-site inspections perfunctory, or even collude with enterprises to “greenwashing” [19]. The research by Koupo et al. reveals that when local government supervision is absent, “irregularities” by certification institutions become an evolutionarily stable strategy [20]; while Wang Xin’s game model confirms that an increase in the strictness of certification institutions’ reviews can effectively promote green technological innovation by enterprises [21]. With the continuous in-depth application of game theory in environmental governance, scholars have gradually realized that the process of environmental regulation is essentially a strategic interaction process involving multiple actors, multiple objectives, and multiple constraints. The behavioral choices of governments, enterprises, certification institutions, and consumers in voluntary environmental regulation are not only driven by their own interests and cost-benefit calculations but also influenced by the strategic changes of other participants. Although traditional fully rational game models can analyze the optimal decisions of actors in a static framework, they often assume complete information and full rationality, which is at odds with the limited cognition, information asymmetry, and behavioral constraints of actors in real environmental governance. In contrast, the evolutionary game model is more suitable for the research needs of voluntary environmental regulation in the following aspects: (1) Limited rationality assumption: The evolutionary game assumes that the rationality of the subjects is limited, and their strategy updates rely on historical experience and benefit comparisons, which can better reflect the gradual and imperfect nature of decision-making in reality. (2) Dynamic strategy evolution: This model can depict the strategy adjustments and evolution paths of multiple subjects in long-term repeated interactions, making it suitable for analyzing the impact of policy incentives, market feedback, and changes in social preferences on strategies. (3) Multi-agent interaction: The evolutionary game framework can be extended to multi-party participation scenarios, not only analyzing the bilateral relationship between enterprises and the government, but also simultaneously incorporating certification institutions and consumers, thus forming a system analysis of multi-party collaboration or competition. (4) Stability analysis: By solving the evolutionary stable strategy (ESS) of the system, the long-term equilibrium conditions and stability of high-quality participation can be identified, providing predictive and guiding significance for policy design. Therefore, the research framework based on evolutionary games not only can explain the behavioral logic of multiple subjects in voluntary environmental regulation from a theoretical perspective, but also can test in simulations whether high-quality participation mechanisms can be formed and maintained stably under different policy interventions and market scenarios. This theoretical foundation provides a solid support for the construction of a four-party evolutionary game model of “local governments - enterprises - certification institutions - consumers” in this paper.

Current research still has deficiencies in several aspects: First, the research content mainly focuses on the elaboration of the connotation of voluntary environmental regulations and the summary of participation motives, but lacks a clear definition of the connotation of “high-quality participation” and has not systematically explored its influencing factors. Second, the research methods mostly emphasize macro static evaluations such as difference-in-differences, propensity score matching, and Tobit, and rarely delve into the multi-subject interaction relationships and internal evolution mechanisms during the participation process of voluntary environmental regulations from a micro perspective. Third, the analysis of research objects is relatively one-sided, and there are few systematic studies that place multiple subjects such as local governments, enterprises, certification institutions, and consumers in a unified analytical framework, and even fewer comprehensive analytical models that cover key external constraints. Against this backdrop, this paper makes a breakthrough improvement to the existing analytical framework and game modeling. Previous game analyses of voluntary environmental regulations mostly adopted a three-party framework of “government - enterprise - public”, treating certification institutions as auxiliary enforcement parties or external conditions, failing to reveal their strategic position and behavioral logic in the effectiveness of regulations. This has led to two core theoretical gaps: first, the lack of shaping and characterization of certification institutions as independent game players with autonomous strategic choices; second, the neglect of the endogenous coupling relationship between “certification quality - market trust - participation performance”. To fill these gaps, this paper, based on clarifying the multi-dimensional connotation of high-quality participation, for the first time constructs an evolutionary game model covering four types of subjects: local government – enterprise – certification institution – consumer, and introduces the central ecological and environmental protection inspection and media supervision as external constraints into the system. This enables a dynamic depiction of the regulatory effects and long-term evolutionary paths of strategic interactions among multiple subjects, achieving a conceptual leap from the traditional three-party analysis to a full-chain multi-party collaborative game. Methodologically, this paper combines evolutionary game theory with numerical simulation to systematically compare the relative importance and endogenous mechanisms of variables such as government subsidies, penalty intensity, and media exposure in driving high-quality participation. The research results show that the compliance of certification institutions is the core factor in achieving high-quality participation and maintaining stable equilibrium. This finding not only fills the research gap on the independence and behavioral motivation mechanism of certification institutions but also expands the application boundary of evolutionary game theory in the field of environmental governance, making the model closer to the real operational logic of voluntary environmental regulations. Overall, this paper achieves multiple innovations in subject composition, introduction of external constraints, and revelation of dynamic mechanisms. It not only surpasses the traditional three-party or simplified stakeholder models at the conceptual level but also provides solid theoretical support and simulation-verified empirical evidence for the precise design of policy tools, optimization of institutional arrangements, and stimulation of green technological innovation. It has significant academic value and practical significance for promoting the high-quality operation and sustainable development of voluntary environmental regulations.

## 2 Evolutionary game theory analysis

### 2.1 Implementation process and interactive relationship

**Fig 1:**
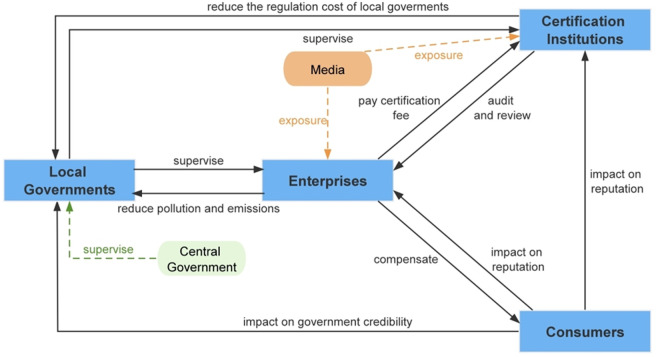
Implementation process and interactive relationship of relevant subjects.

In pursuit of profit maximization, enterprises will pay attention to the requirements of ISO14001 environmental management system certification, prepare and submit materials for certification, and strategically select the certification institutions in order to successfully pass the certification. Certification institutions will review the information and send out the certification qualification mark, as well as supervise the environmental governance of enterprises during the three-year validity period. In this process, certification institutions may violate certification rules and make profits by lowering audit requirements and trading certificates in violation of the law. Local governments will selectively implement policies related to voluntary environmental regulation for the sake of local economic development and improved environmental performance. Consumers will be concerned about the state of the environment and the quality of green products, but may choose not to be concerned due to the difficulty of speaking out. In addition, the whole interactive process will be influenced by the central supervision and the media, the central eco-environmental supervision to punish the negligent local government, the media to all kinds of negative environmental behaviors to objectively and truthfully expose and report. Implementation process and interactive relationship of relevant subjects is shown in Fig 1.

### 2.2 Basic assumptions

According to the evolutionary game theory, the following three basic premises must be met before constructing and establishing a four-party evolutionary game model.

**P1:** As parties of the game, local governments, enterprises, certification institutions and consumers are limited rationality. The goal of the local governments is to maximize local interests, the goal of the enterprises is to maximize profits, the goal of the certification institutions is to maximize revenue, the goal of the consumers is to buy more suitable products and safeguard their own rights and interests. The strategic choices of each party are not in any order and are carried out at the same time.

**P2**: Each of the four game parties has two strategies to choose from in their strategy set: local governments {active promotion, negative promotion}; enterprises {qualified applications, unqualified applications}; certification institutions {legal certification, illegal certification}; consumers {strong concern, weak concern}.

**P3**: The probability that local governments choose the active promotion strategy is x(0≤x≤1), the probability of negative promotion strategy is 1−x. The probability that enterprises choose the qualified applications strategy is y(0≤y≤1), the probability of unqualified applications strategy is 1−y. The probability that certification institutions choose the legal certification strategy is  z(0≤z≤1), the probability of illegal certification strategy is 1−z. The probability that consumers choose the strong concern strategy is w (0≤w≤1), the probability of weak concern strategy is 1−w.

**Fig 2:**
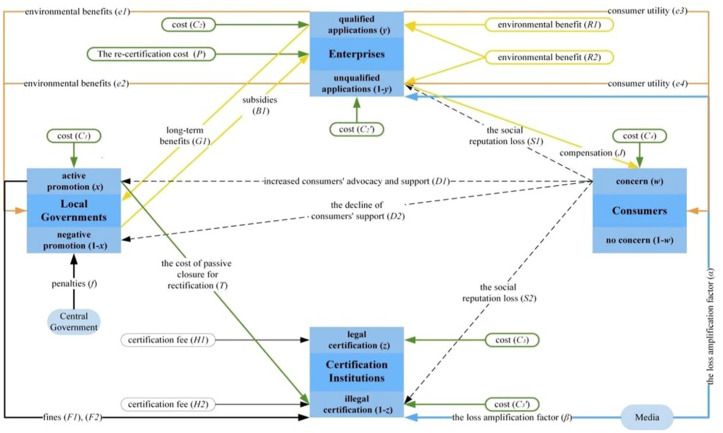
The relationship of the subjects and parameters.

On the basis of satisfying the above premise, combined with the specific implementation process and the basic interactive relationship of voluntary environmental regulation in China, the model assumptions are put forward and the meaning of each parameter is shown in Table 1, and the relationship of subjects and parameters is shown in Fig 2.

**Table 1:** The symbolic and meanings of the parameters.

Symbolic	Meaning
$C_{\text{1}}$	The active promotion cost of local government
G1	Long-term benefits of local government
D1	Increased consumers’ advocacy and support
f	Penalties imposed by the central government on local governments when they fail to perform their duties
D2	The loss of passive implementation reflected as the decline of consumers’ support
$C_{\text{2}}$	The cost of enterprises meeting the conditions to apply
B1	Subsidies to certified enterprises
R1	Environmental benefit of certified enterprises when legal certification
$C_{\text{2}}^{\prime }$	The cost of enterprises not meeting the conditions to apply
R2	Environmental benefit of certified enterprises when illegal certification
S1	The social reputation loss of enterprises
α	The loss amplification factor of the enterprises under the media exposure
P	The re-certification cost of enterprises
$C_{\text{3}}$	Certification cost when certification institutions choose the “legal certification” strategy
H1	Enterprise’ certification fee when certification institutions choose the “legal certification” strategy
$C_{\text{3}}^{\prime }$	Certification cost when certification institutions choose the “illegal certification” strategy
H2	Enterprise’ certification fee when certification institutions choose the “illegal certification” strategy
F1	Fines to the illegal certification institutions when enterprises meet the certification conditions
F2	Fines to the illegal certification institutions when enterprises do not meet the certification conditions
T	The cost of passive closure for rectification of certification institutions
S2	The social reputation loss of certification institutions
β	The loss amplification factor of the certification institutions under media exposure
$C_{\text{4}}$	The cost of consumers’ concern
J	Enterprises’ compensation to consumers
e1	Increased environmental benefits of local government
e2	Decreased environmental benefits of local government
$E_{\text{1}}$	Increased consumer utility when enterprises meet the certification conditions
$E_{\text{2}}$	Decreased consumer utility when enterprises do not meet the certification conditions

**H1:** When local governments adopt the “active promotion” strategy, it means that local governments encourage more enterprises to participate in voluntary environmental regulation by the form of both subsidies and penalties. If enterprises are certified, local governments will reward them with B1. At this time, the active promotion cost of local government is $C_{\text{1}}$, referring to the various costs of human resources and materials spent by local departments, which is shown that the local market supervision authority is responsible for organizing the declaration of subsidies, the Ministry of Finance and other departments are responsible for the disbursement of funds, and the Market Supervision Authority and the Bureau of Ecology and Environment is responsible for the supervision. Local governments receive long-term benefits G1, including reduced environmental regulatory costs, improved environmental conditions and high-quality economic development. When consumers are concerned, local governments obtain their support as D1. The local environmental benefits increase by e1 when enterprises fulfil the conditions to apply, and decrease to e2 conversely.

When local governments adopt the “negative promotion” strategy, it means that the efforts to push ahead with voluntary environmental regulation are very weak. For the convenience of calculation, it is assumed that the cost of local governments is 0 and there are no subsidies or penalties. The central government carries out environmental supervision on the environmental governance status of local governments, and penalties f on local governments’ failing in their duties when consumers are concerned. When consumers are concerned, the loss of local governments’ passive implementation of voluntary environmental regulation is D2, reflected as the decline of consumers’ support to local governments.

**H2**: When enterprises choose the “qualified applications” strategy, the application cost is $C_{\text{2}}$, which specifically includes the cost of learning the requirements of the ISO14001 standard, carrying out internal training, formulating environmental policy documents, carrying out environmental management related activities, obtaining proof of compliance by environmental authorities during the past one year, compliance of pollutants with emission standards, environmental monitoring reports, environmental acceptance reports, EIA reports, three simultaneous acceptance, etc., as well as passing internal audits and management reviews, etc. Enterprises can obtain local government subsidies B1 and environmental benefits R1 which is expressed as lower pollutant emissions, lower probability of penalties and so on after certification.

When enterprises choose the “unqualified applications” strategy, it means that they only carry out part of the activities, fulfil part of the requirements and the cost is $C_{\text{2}}^{\prime }(C_{\text{2}}>C_{\text{2}}^{\prime }>\text{0})$. When certification institutions conduct legal certification, these enterprises can not pass the certification and can not get the benefits. On the contrary, when certification institutions conduct illegal certification, these enterprises can pass the certification and obtain environmental benefits R2(R1>R2>0), and at the same time, the green advantages of the enterprises that meet the application conditions will be reduced, and the benefits will be reduced to R2. When enterprises obtain certificates under non-compliant circumstances, they will face reputational losses S1 in society once they are exposed by the media with a probability of m and are under intense consumer scrutiny. This loss is mainly reflected in the decline of the credibility of the certificates obtained by the enterprise and the reduction of consumers’ trust in it. The loss amplification factor of enterprises is α(α>1) when local governments actively implement voluntary environmental regulation and the media actively expose. Once the illegal certification is found, the certified enterprise needs to re-certify and pay additional time, human and financial costs P.

**H3**: When certification institutions choose the “legal certification” strategy, it means that certification institutions have well-established and complex procedures in the certification process, with the certification cost $C_{\text{3}}$ and enterprise’ certification fee H1.

When the “illegal certification” strategy is chosen, it means that certification institutions have a lax certification process and the lower standard of scrutiny, such as reducing the audit time, forging certification materials, employing unregistered certifiers, and failing to conduct supervision and audit on schedule, etc. At this time, the cost of certification reduces to $C_{\text{3}}^{\prime }(C_{\text{3}}>C_{\text{3}}^{\prime }>\text{0})$ and enterprise’ certification fee increases to H2 (H2>H1>0). Under media exposure, the certification institutions will be fined F1 when enterprises meet the certification conditions, and F2 (0<F1<F2) when enterprises do not meet the certification conditions, the cost of passive closure for rectification T is generated, and the loss of social reputation of certification institutions is S2 when consumers are concerned. The loss amplification factor of illegal certification institutions is β(β>1) when local governments actively implement voluntary environmental regulation and the media actively expose.

**H4**: When consumers choose the “strong concern” strategy, it means that consumers have green awareness and are highly concerned on environmental issues and enterprises’ environmental performance. In order to purchase a more desirable green product, consumers will gather price information, compare qualities, confirm quality and safety, etc., and thus generate cost $C_{\text{4}}$. When enterprises meet the certification conditions, consumer utility increases e3, and vice versa, consumer utility decreases e4. When enterprises do not meet the certification conditions, consumer rights and interests are damaged, and various media exposure platforms help consumers defend their rights, at this time, regardless of whether local governments actively implement voluntary environmental regulations, enterprises will be ordered to give consumers compensation J. When consumers adopt the “weak concern” strategy, it indicates that their overall sensitivity to environmental issues is relatively low and their level of attention is limited. They do not actively assess or compare the green and environmentally friendly features of products. Therefore, their supervisory activities during the purchase and usage process are almost non-existent, and the related costs can be regarded as 0.

### 2.3 Model construction

According to the above basic assumptions, the payment matrix of the four-party game model of voluntary environmental regulation can be obtained as Table 2.

**Table 2:** Payment matrix.

		Certification institutions legal certification z		Certification institutions illegal certification 1−z	
		Consumers concern w	Consumers no concern 1−w	Consumers concern w	Consumers no concern 1−w
Local governments active promotion x	Enterprises qualified applications y	$a_{\text{1}}=-C_{\text{1}}+G\text{1}-B\text{1}+e\text{1}+D\text{1}$ $a_{\text{2}}=-C_{\text{2}}+B\text{1}+R\text{1}-H\text{1}$ $a_{\text{3}}=-C_{\text{3}}+H\text{1}$ $a_{\text{4}}=-C_{\text{4}}+e\text{3}$	$a_{\text{5}}=-C_{\text{1}}+G\text{1}-B\text{1}+e\text{1}$ ${\text{a}}_{\text{6}}=-C_{\text{2}}+B\text{1}+R\text{1}-H\text{1}$ $a_{\text{7}}=-C_{\text{3}}+H\text{1}$ $a_{\text{8}}=e\text{3}$	$a_{\text{9}}=-C_{\text{1}}+G\text{1}-B\text{1}+e\text{1}+D\text{1}+\beta F\text{1}$ $a_{\text{1}\text{0}}=-C_{\text{2}}+B\text{1}+R\text{2}-H\text{2}-P$ $a_{\text{1}\text{1}}=-C_{\text{3}}^{\prime }+H\text{2}-\beta F\text{1}-\beta S\text{2}$ $a_{\text{1}\text{2}}=-C_{\text{4}}+e\text{3}$	${\text{a}}_{\text{13}}=-C_{\text{1}}+G\text{1}-B\text{1}+e\text{1}+\beta F\text{1}$ $a_{\text{1}\text{4}}=-C_{\text{2}}+B\text{1}+R\text{2}-H\text{2}-P$ $a_{\text{1}\text{5}}=-C_{\text{3}}^{\prime }+H\text{2}-\beta F\text{1}$ $a_{\text{1}\text{6}}=e\text{3}$
	Enterprises unqualified applications 1−y	$a_{\text{1}\text{7}}=-C_{\text{1}}-e\text{2}$ $a_{\text{1}\text{8}}=-C_{\text{2}}^{\prime }-H\text{1}-J-\alpha S\text{1}$ $a_{\text{1}\text{9}}=-C_{\text{3}}+H\text{1}$ $a_{\text{2}\text{0}}=-C_{\text{4}}-e\text{4}+$ *J*	$a_{\text{2}\text{1}}=-C_{\text{1}}-e\text{2}$ $a_{\text{2}\text{2}}=-C_{\text{2}}^{\prime }-H\text{1}$ $a_{\text{2}\text{3}}=-C_{\text{3}}+H\text{1}$ $a_{\text{2}\text{4}}=-e\text{4}$	$a_{\text{2}\text{5}}=-C_{\text{1}}-B\text{1}-e\text{2}+\beta F\text{2}$ $a_{\text{2}\text{6}}=-C_{\text{2}}^{\prime }+B\text{1}+R\text{2}-\alpha S\text{1}-H\text{2}-J-P$ $a_{\text{2}\text{7}}=-C_{\text{3}}^{\prime }+H\text{2}-\beta F\text{2}-\beta S\text{2}-\beta T$ $a_{\text{2}\text{8}}=-C_{\text{4}}-e\text{4}+J$	$a_{\text{2}\text{9}}=-C_{\text{1}}-B\text{1}-e\text{2}\;+\beta F\text{2}$ $a_{\text{3}\text{0}}=-C_{\text{2}}^{\prime }+B\text{1}+R\text{2}-H\text{2}-P$ $a_{\text{3}\text{1}}=-C_{\text{3}}^{\prime }+H\text{2}-\beta F\text{2}-\beta T$ $a_{\text{3}\text{2}}=-e\text{4}$
Local governments negative promotion 1−x	Enterprises qualified applications y	$a_{\text{3}\text{3}}=e\text{1}$ $a_{\text{3}\text{4}}=-C_{\text{2}}+R\text{1}-H\text{1}$ $a_{\text{3}\text{5}}=-C_{\text{3}}+H\text{1}$ $a_{\text{3}\text{6}}=-C_{\text{4}}+e\text{3}$	$a_{\text{3}\text{7}}=e\text{1}$ $a_{\text{3}\text{8}}=-C_{\text{2}}+R\text{1}-H\text{1}$ $a_{\text{3}\text{9}}=-C_{\text{3}}+H\text{1}$ $a_{\text{4}\text{0}}=e\text{3}$	$a_{\text{4}\text{1}}=e\text{1}$ $a_{\text{4}\text{2}}=-C_{\text{2}}+R\text{2}-H\text{2}$ $a_{\text{4}\text{3}}=-C_{\text{3}}^{\prime }+H\text{2}-S\text{2}$ $a_{\text{4}\text{4}}=-C_{\text{4}}+e\text{3}$	$a_{\text{4}\text{5}}=e\text{1}$ $a_{\text{4}\text{6}}=-C_{\text{2}}+R\text{2}-H\text{2}$ $a_{\text{4}\text{7}}=-C_{\text{3}}^{\prime }+H\text{2}$ $a_{\text{4}\text{8}}=e\text{3}$
	Enterprises unqualified applications 1−y	$a_{\text{4}\text{9}}=-e\text{2}-D\text{2}$ $a_{\text{5}\text{0}}=-C_{\text{2}}^{\prime }-H\text{1}-J-S\text{1}$ $a_{\text{5}\text{1}}=-C_{\text{3}}+H\text{1}$ $a_{\text{5}\text{2}}=-C_{\text{4}}-e\text{4}+J$	$a_{\text{5}\text{3}}=-e\text{2}$ $a_{\text{5}\text{4}}=-C_{\text{2}}^{\prime }-H\text{1}$ $a_{\text{5}\text{5}}=-C_{\text{3}}+H\text{1}$ $a_{\text{5}\text{6}}=-e\text{4}$	$a_{\text{5}\text{7}}=-e\text{2}-f-D\text{2}$ $a_{\text{5}\text{8}}=-C_{\text{2}}^{\prime }+R\text{2}-H\text{2}-J-S\text{1}$ $a_{\text{5}\text{9}}=-C_{\text{3}}^{\prime }+H\text{2}-S\text{2}$ $a_{\text{6}\text{0}}=-C_{\text{4}}-e\text{4}+J$	$a_{\text{6}\text{1}}=-e\text{2}-f$ $a_{\text{6}\text{2}}=-C_{\text{2}}^{\prime }+R\text{2}-H\text{2}$ $a_{\text{6}\text{3}}=-C_{\text{3}}^{\prime }+H\text{2}$ $a_{\text{6}\text{4}}=-e\text{4}$

### 2.4 Model derivation and equilibrium points stability analysis

On the basis of the above model setting, suppose the fitness of local governments to choose the active promotion strategy is v11, the fitness of the negative promotion strategy is v12, and v1 is the average fitness, then:


$$\begin{array}{c} v\text{1}\text{1}=yzwa_{\text{1}}+yz(\text{1}-w)a_{\text{5}}+y(\text{1}-z)wa_{\text{9}}+y(\text{1}-z)(\text{1}-w)a_{\text{1}\text{3}}+(\text{1}-y)zwa_{\text{1}\text{7}}+(\text{1}-y)z(\text{1}-w)a_{\text{2}\text{1}}\\ +(\text{1}-y)(\text{1}-z)wa_{\text{2}\text{5}}+(\text{1}-y)(\text{1}-z)(\text{1}-w)a_{\text{2}\text{9}} \end{array}$$ (1)

$$\begin{array}{c} v\text{1}\text{2}=yzwa_{\text{3}\text{3}}+yz(\text{1}-w)a_{\text{3}\text{7}}+y(\text{1}-z)wa_{\text{4}\text{1}}+y(\text{1}-z)(\text{1}-w)a_{\text{4}\text{5}}+(\text{1}-y)zwa_{\text{4}\text{9}}+(\text{1}-y)z(\text{1}-w)a_{\text{5}\text{3}}\\ +(\text{1}-y)(\text{1}-z)wa_{\text{5}\text{7}}+(\text{1}-y)(\text{1}-z)(\text{1}-w)a_{\text{6}\text{1}} \end{array}$$ (2)

v1=xv11+(1−x)v12 (3)

According to the Malthusian equation, the quantitative growth rate of the local governments choosing active promotion strategy is equal to the difference between their fitness and the average fitness. Substituting into the corresponding expression of payment value can obtain the replication dynamic equation of the local governments’ active promotion strategy:


$$\begin{array}{c} f(x)=x(v\text{1}\text{1}-v\text{1})=x(x-\text{1})(B\text{1}+C_{\text{1}}-f-\beta F\text{2}-D\text{2}w-B\text{1}z-G\text{1}y+fy+fz-\beta F\text{1}y+\beta F\text{2}y+\;\beta F\text{2}z\\ -D\text{1}wy+D\text{2}wy+B\text{1}yz-fyz+\beta F\text{1}yz-\beta F\text{2}yz) \end{array}$$ (4)

Similarly, the replication dynamic equations of the other three parties can be obtained separately, and the four-dimensional dynamic system of the high-quality participation model of voluntary environmental regulation can be obtained simultaneously as follows:


$$\begin{array}{c} fx=x(x-\text{1})(B\text{1}+C_{\text{1}}-f-\beta F\text{2}-D\text{2}w-B\text{1}z-G\text{1}y+fy+fz-\beta F\text{1}y+\beta F\text{2}y+\beta F\text{2}z-D\text{1}wy+D\text{2}wy\\ +B\text{1}yz-fyz+\beta F\text{1}yz-\beta F\text{2}yz)\\ fy=y(y-\text{1})({-C}_{\text{2}}^{\prime }+C_{\text{2}}-Jw-S\text{1}w-R\text{1}z-B\text{1}xz\;+S\text{1}wx-\alpha S\text{1}wx) \end{array}$$ (5)

$$\begin{array}{c} fz=z(z-\text{1})({-C}_{\text{3}}^{\prime }+C_{\text{3}}-H\text{1}+H\text{2}-S\text{2}w-\beta F\text{2}x-\beta Tx+S\text{2}wx-\beta S\text{2}wx+\beta Txy-\beta F\text{1}xy+\beta F\text{2}xy)\\ fw=w(w-\text{1})(C_{\text{4}}-J+Jy) \end{array}$$

Let f(x)=0, f(y)=0, f(z)=0, f(w)=0, solve the system to get a total of 16 pure strategy equilibrium points (0,0,0,0), (0,0,0,1), …, (1,1,1,1). The Jacobian matrix *J* corresponding to the system can be obtained by calculating the first order partial derivative of Eq. (5):


$$J=[\begin{array}{cccc} \frac{\partial \dot{x}}{\partial x} & \frac{\partial \dot{x}}{\partial y} & \frac{\partial \dot{x}}{\partial z} & \frac{\partial \dot{x}}{\partial w}\\ \frac{\partial \dot{y}}{\partial x} & \frac{\partial \dot{y}}{\partial y} & \frac{\partial \dot{y}}{\partial z} & \frac{\partial \dot{y}}{\partial w}\\ \frac{\partial \dot{z}}{\partial x} & \frac{\partial \dot{z}}{\partial y} & \frac{\partial \dot{z}}{\partial z} & \frac{\partial \dot{z}}{\partial w}\\ \frac{\partial \dot{w}}{\partial x} & \frac{\partial \dot{w}}{\partial y} & \frac{\partial \dot{w}}{\partial z} & \frac{\partial \dot{w}}{\partial w} \end{array}]$$ (6)

Substitute the 16 pure strategy equilibrium points into Eq. (6), the eigenvalues corresponding to each pure strategy equilibrium point of the four-dimensional dynamical system can be obtained. According to the first method of Lyapunov, when all the eigenvalues of matrix J have negative real parts, it can be determined that the system is asymptotically stable at this point, and its corresponding strategy combination is the evolutionary stable strategy of the system. After analyzing one by one, it can be determined that there are 7 stable points among the 16 equilibrium points as shown in Table 3.

**Table 3:** Stability of each equilibrium point of the system.

Equilibrium point	Eigenvalues					Ideality
	λ1	λ2	λ3	λ4	Situation	
$E_{\text{1}}(\text{0},\text{0},\text{0},\text{0})$	$-C_{\text{1}}-B\text{1}+f+\beta F\text{2}$	$C_{\text{2}}^{\prime }-C_{\text{2}}$	$C_{\text{3}}^{\prime }-C_{\text{3}}+H\text{1}-H\text{2}$	$J-C_{\text{4}}$	* - - *	Non-ideal
$E_{\text{2}}(\text{0},\text{0},\text{0},\text{1})$	$-C_{\text{1}}-B\text{1}+D\text{2}+f+\beta F\text{2}$	$C_{\text{2}}^{\prime }-C_{\text{2}}+S\text{1}+J$	$C_{\text{3}}^{\prime }-C_{\text{3}}+H\text{1}-H\text{2}+S\text{2}$	$C_{\text{4}}-J$	* * * *	Non-ideal
$E_{\text{3}}(\text{0},\text{0},\text{1},\text{0})$	$-C_{\text{1}}$	$C_{\text{2}}^{\prime }-C_{\text{2}}+R\text{1}$	$C_{\text{3}}-C_{\text{3}}^{\prime }-H\text{1}+H\text{2}$	$J-C_{\text{4}}$	- * + *	–
$E_{\text{4}}(\text{0},\text{0},\text{1},\text{1})$	$D\text{2}-C_{\text{1}}$	$C_{\text{2}}^{\prime }-C_{\text{2}}+R\text{1}+S\text{1}+J$	$C_{\text{3}}-C_{\text{3}}^{\prime }-H\text{1}+H\text{2}-S\text{2}$	$C_{\text{4}}-J$	* * * *	Relatively ideal
$E_{\text{5}}(\text{0},\text{1},\text{0},\text{0})$	$-C_{\text{1}}-B\text{1}+G\text{1}+\beta F\text{1}$	$C_{\text{2}}-C_{\text{2}}^{\prime }$	$C_{\text{3}}^{\prime }-C_{\text{3}}+H\text{1}-H\text{2}$	$-C_{\text{4}}$	+ + - -	–
$E_{\text{6}}(\text{0},\text{1},\text{0},\text{1})$	$-C_{\text{1}}-B\text{1}+G\text{1}+D\text{1}+\beta F\text{1}$	$C_{\text{2}}-C_{\text{2}}^{\prime }-S\text{1}-J$	$C_{\text{3}}^{\prime }-C_{\text{3}}+H\text{1}-H\text{2}+S\text{2}$	$C_{\text{4}}$	+ * * +	–
$E_{\text{7}}(\text{0},\text{1},\text{1},\text{0})$	$G\text{1}-B\text{1}-C_{\text{1}}$	$C_{\text{2}}-C_{\text{2}}^{\prime }-R\text{1}$	$C_{\text{3}}-C_{\text{3}}^{\prime }-H\text{1}+H\text{2}$	$-C_{\text{4}}$	+ * + -	–
$E_{\text{8}}(\text{0},\text{1},\text{1},\text{1})$	$D\text{1}-C_{\text{1}}-B\text{1}+G\text{1}$	$C_{\text{2}}-C_{\text{2}}^{\prime }-R\text{1}-S\text{1}-J$	$C_{\text{3}}-C_{\text{3}}^{\prime }-H\text{1}+H\text{2}-S\text{2}$	$C_{\text{4}}$	+ * * +	–
$E_{\text{9}}(\text{1},\text{0},\text{0},\text{0})$	$B\text{1}+C_{\text{1}}-f-\beta F\text{2}$	$C_{\text{2}}^{\prime }-C_{\text{2}}$	$C_{\text{3}}^{\prime }-C_{\text{3}}+H\text{1}-H\text{2}+\beta F\text{2}+\beta T$	$J-C_{\text{4}}$	* - * *	Non-ideal
$E_{\text{1}\text{0}}(\text{1},\text{0},\text{0},\text{1})$	$B\text{1}+C_{\text{1}}-D\text{2}-f-\beta F\text{2}$	$C_{\text{2}}^{\prime }-C_{\text{2}}+\alpha S\text{1}+J$	$C_{\text{3}}^{\prime }-C_{\text{3}}+H\text{1}-H\text{2}+\beta F\text{2}+\beta S\text{2}+\beta T$	$C_{\text{4}}-J$	* * * *	Non-ideal
$E_{\text{1}\text{1}}(\text{1},\text{0},\text{1},\text{0})$	$C_{\text{1}}$	$B\text{1}-C_{\text{2}}+C_{\text{2}}^{\prime }+R\text{1}$	$C_{\text{3}}-C_{\text{3}}^{\prime }-H\text{1}+H\text{2}-\beta F\text{2}-\beta T$	$J-C_{\text{4}}$	+ * * *	–
$E_{\text{1}\text{2}}(\text{1},\text{0},\text{1},\text{1})$	$C_{\text{1}}-D\text{2}$	$B\text{1}-C_{\text{2}}+C_{\text{2}}^{\prime }+R\text{1}+\alpha S\text{1}+J$	$C_{\text{3}}-C_{\text{3}}^{\prime }-H\text{1}+H\text{2}-\beta F\text{2}-\beta S\text{2}-\beta T$	$C_{\text{4}}-J$	* * * *	Relatively ideal
$E_{\text{1}\text{3}}(\text{1},\text{1},\text{0},\text{0})$	$B\text{1}+C_{\text{1}}-G\text{1}-\beta F\text{1}$	$C_{\text{2}}-C_{\text{2}}^{\prime }$	$C_{\text{3}}^{\prime }-C_{\text{3}}+H\text{1}-H\text{2}+\beta F\text{1}$	$-C_{\text{4}}$	- + * -	–
$E_{\text{1}\text{4}}(\text{1},\text{1},\text{0},\text{1})$	$B\text{1}+C_{\text{1}}-D\text{1}-G\text{1}-\beta F\text{1}$	$C_{\text{2}}-C_{\text{2}}^{\prime }-J-\alpha S\text{1}$	$C_{\text{3}}^{\prime }-C_{\text{3}}+H\text{1}-H\text{2}+\beta F\text{1}+\beta S\text{2}$	$C_{\text{4}}$	- * * +	–
$E_{\text{1}\text{5}}(\text{1},\text{1},\text{1},\text{0})$	$B\text{1}+C_{\text{1}}-G\text{1}$	$C_{\text{2}}-B\text{1}-C_{\text{2}}^{\prime }-R\text{1}$	$C_{\text{3}}-C_{\text{3}}^{\prime }-H\text{1}+H\text{2}-\beta F\text{1}$	$-C_{\text{4}}$	- * * -	Ideal
$E_{\text{1}\text{6}}(\text{1},\text{1},\text{1},\text{1})$	$B\text{1}+C_{\text{1}}-D\text{1}-G\text{1}$	$C_{\text{2}}-B\text{1}-C_{\text{2}}^{\prime }-J-R\text{1}-\alpha S\text{1}$	$C_{\text{3}}-C_{\text{3}}^{\prime }-H\text{1}+H\text{2}-\beta F\text{1}-\beta S\text{2}$	$C_{\text{4}}$	- - * +	–

* indicates that the positive and negative are uncertain.

Therefore $E_{\text{3}}$, $E_{\text{5}}$*-*$E_{\text{8}}$, $E_{\text{1}\text{1}}$, $E_{\text{1}\text{3}}$, $E_{\text{1}\text{4}}$ and $E_{\text{1}\text{6}}$ cannot be the evolutionary stabilization point (ESS) of the system in any case, so the discussion is only for the equilibrium point $E_{\text{1}}(\text{0},\text{0},\text{0},\text{0})$, $E_{\text{2}}(\text{0},\text{0},\text{0},\text{1})$, $E_{\text{4}}(\text{0},\text{0},\text{1},\text{1})$, $E_{\text{9}}(\text{1},\text{0},\text{0},\text{0})$, $E_{\text{1}\text{0}}(\text{1},\text{0},\text{0},\text{1})$, $E_{\text{1}\text{2}}(\text{1},\text{0},\text{1},\text{1})$, $E_{\text{1}\text{5}}(\text{1},\text{1},\text{1},\text{0})$.

#### 2.4.1 Stability analysis of equilibrium point $E_{1}\left(0,0,0,0\right)$.

The point $E_{\text{1}}(\text{0},\text{0},\text{0},\text{0})$ is asymptotically stable when $-C_{\text{1}}-B\text{1}+f+\beta F\text{2}<\text{0}$, $C_{\text{2}}^{\prime }-C_{\text{2}}<\text{0}$, $C_{\text{3}}^{\prime }-C_{\text{3}}+H\text{1}-H\text{2}<\text{0}$ and $J-C_{\text{4}}<\text{0}$. Four parties respond negatively to voluntary environmental regulation, the central government has difficulties in implementing the task of ecological civilization construction, and the high-quality participation level of voluntary environmental regulation in this region is low, which is an undesirable stable equilibrium point.

#### 2.4.2 Stability analysis of equilibrium point $E_{2}\left(0,0,0,1\right)$.

The point $E_{\text{2}}(\text{0},\text{0},\text{0},\text{1})$ is asymptotically stable when $-C_{\text{1}}-B\text{1}+D\text{2}+f+\beta F\text{2}<\text{0}$, $C_{\text{2}}^{\prime }-C_{\text{2}}+S\text{1}+J<\text{0}$, $C_{\text{3}}^{\prime }-C_{\text{3}}+H\text{1}-H\text{2}+S\text{2}<\text{0}$ and $C_{\text{4}}-J<\text{0}$. Compared with the previous situation, consumers are concerned about the environmental governance level of local governments, pollution control level of enterprises and status of the certification institutions, but fail to change the strategic choices of other parties, indicating that consumers’ concern can not play a decisive role in enhancing the high-quality participation in voluntary environmental regulation. The high-quality participation level of voluntary environmental regulation in this region is low, which is an undesirable stable equilibrium point.

#### 2.4.3 Stability analysis of equilibrium point $E_{4}\left(0,0,1,1\right)$.

The point $E_{\text{4}}(\text{0},\text{0},\text{1},\text{1})$ is asymptotically stable when $D\text{2}-C_{\text{1}}<\text{0}$, $C_{\text{2}}^{\prime }-C_{\text{2}}+R\text{1}+S\text{1}+J<\text{0}$, $C_{\text{3}}-C_{\text{3}}^{\prime }-H\text{1}+H\text{2}-S\text{2}<\text{0}$ and $C_{\text{4}}-J<\text{0}$. Compared with the previous situation, the certification institutions choose the legal certification, strictly review the applicant materials, actively carry out on-site audits and other initiatives, and supervise the certified enterprises during the validity period, ensure the effectiveness and compliance of the certification, and reduce the regulatory costs of local governments on enterprises. The high-quality participation level of voluntary environmental regulation in this region is improved, which is an ideal stable equilibrium point.

#### 2.4.4 Stability analysis of equilibrium point $E_{9}\left(1,0,0,0\right)$.

The point $E_{\text{9}}(\text{1},\text{0},\text{0},\text{0})$ is asymptotically stable when $B\text{1}+C_{\text{1}}-f-\beta F\text{2}<\text{0}$, $C_{\text{2}}^{\prime }-C_{\text{2}}<\text{0}$, $C_{\text{3}}^{\prime }-C_{\text{3}}+H\text{1}-H\text{2}+\beta F\text{2}+\beta T<\text{0}$ and $J-C_{\text{4}}<\text{0}$. Local governments provide subsidies to certified enterprises, although the financial pressure of enterprises can be alleviated to a certain extent, but the qualified applications cost of enterprises is relatively high. Especially for small and medium-sized enterprises, the high cost incurred during certification may easily lead them into financing difficulties and affect the normal operation of enterprises. At the same time, even if faced with government penalties, certification institutions will choose illegal certification under the temptation of interests. The high-quality participation level of voluntary environmental regulation in this region is low, which is an undesirable stable equilibrium point.

#### 2.4.5 Stability analysis of equilibrium point $E_{10}\left(1,0,0,1\right)$.

The point ${\text{E}}_{\text{10}}(\text{1},\text{0},\text{0},\text{1})$ is asymptotically stable when $B\text{1}+C_{\text{1}}-D\text{2}-f-\beta F\text{2}<\text{0}$, $C_{\text{2}}^{\prime }-C_{\text{2}}+\alpha S\text{1}+J<\text{0}$, $C_{\text{3}}^{\prime }-C_{\text{3}}+H\text{1}-H\text{2}+\beta F\text{2}+\beta S\text{2}+\beta T<\text{0}$ and $C_{\text{4}}-J<\text{0}$. Compared with the previous situation, consumers pay attention to the environmental performance of enterprises, prefer green products with ISO14001 certification mark, and report enterprises that are in violation of the certification. However, from the perspective of cost, enterprises still choose the strategy of unqualified applications, and obtain certificates by paying higher certification fees to illegal certification institutions in order to enjoy green benefits and local government subsidies. The high-quality participation level of voluntary environmental regulation in this region is low, which is an undesirable stable equilibrium point.

#### 2.4.6 Stability analysis of equilibrium point $E_{12}\left(1,0,1,1\right)$.

The point $E_{\text{1}\text{2}}(\text{1},\text{0},\text{1},\text{1})$ is asymptotically stable when $C_{\text{1}}-D\text{2}<\text{0}$, $B\text{1}-C_{\text{2}}+C_{\text{2}}^{\prime }+R\text{1}+\alpha S\text{1}+J<\text{0}$, $C_{\text{3}}-C_{\text{3}}^{\prime }-H\text{1}+H\text{2}-\beta F\text{2}-\beta S\text{2}-\beta T<\text{0}$ and $C_{\text{4}}-J<\text{0}$. At this time, local governments actively promote voluntary environmental regulation in order to avoid the decrease in the degree of consumers’ support for the governments, take the responsibility for improving environmental quality, provide direct financial subsidies to certified enterprises, supervise certification institutions and publish black and white lists. Media exposure further aggravates the losses of illegal certification institutions. Certification institutions will strictly control the certification process in order to avoid the fines, reputation loss, closure and other adverse effects. Consumers will also play an active role for the consideration of reporting rewards, continuously reduce the possibility of unqualified applications together with local governments and certification institutions. The high-quality participation level of voluntary environmental regulation in this region will be improved, which is an desirable stable equilibrium point.

#### 2.4.7 Stability analysis of equilibrium point$E_{15}\left(1,1,1,0\right)$.

The point $E_{\text{1}\text{5}}(\text{1},\text{1},\text{1},\text{0})$ is asymptotically stable when $B\text{1}+C_{\text{1}}-G\text{1}<\text{0}$, $C_{\text{2}}-B\text{1}-C_{\text{2}}^{\prime }-R\text{1}<\text{0}$, $C_{\text{3}}-C_{\text{3}}^{\prime }-H\text{1}+H\text{2}-\beta F\text{1}<\text{0}$ and −C4<0. Active promotion strategy of local governments can directly reduce their regulatory cost for enterprises. Enterprises will actively choose the strategy of qualified applications when the amount of subsidies and environmental benefits are higher than the difference of cost between qualified applications and unqualified applications. The certification institutions tend to choose legal certification strategy when the penalties for illegal certification of certification institutions are higher than their benefits. Therefore, even if consumers do not pay attention to the environmental status and product quality of enterprises, other parties will still choose positive environmental strategies. The high-quality participation level of voluntary environmental regulation in this region will be improved, which is an desirable stable equilibrium point. In this equilibrium, high-quality participation is jointly guaranteed by the proactive strategies of local governments, enterprises, and certification institutions. Regardless of whether consumers pay attention or not, the compliance and environmental protection levels of enterprises can be stably maintained. Due to the effective closed loop formed by local governments’ incentives and supervision, as well as the reputation and penalty constraints of certification institutions, the marginal impact of consumers’ strong attention on the game outcome is extremely small. At the same time, if consumers choose to pay strong attention, they need to bear additional costs, but the effort does not bring substantial improvement in benefits. Under such conditions of cost-benefit asymmetry, consumers rationally choose to pay mild attention, thus forming the characteristic pattern of equilibrium point $E_{\text{1}\text{5}}$, where consumers are in a marginalized role, and their behavior’s decisive role in high-quality participation is weakened by the institutional constraints and incentive mechanisms of other subjects.

In this model, different stable equilibrium points reflect the long-term strategy combinations of multiple actors under voluntary environmental regulations in various scenarios, and the transformation between states is achieved through changes in key parameters. Specifically, $E_{\text{1}}$ and $E_{\text{2}}$, as well as $E_{\text{9}}$ and $E_{\text{1}\text{0}}$, present similar low-quality participation patterns under different consumer concern states and are convertible to each other. $E_{\text{4}}$ and $E_{\text{1}\text{2}}$ both rely on the compliance certification of certification institutions to improve participation levels, with the former representing a scenario where local governments passively promote and the latter representing a scenario where the government actively promotes. At the high-quality participation level, $E_{\text{1}\text{5}}$ represents the optimal state where local governments, enterprises, and certification institutions all adopt proactive strategies. The transformation between different equilibrium points is mainly driven by parameters such as government subsidies, penalty intensity, and media exposure. For instance, increasing the cost of certification institution violations can prompt the system to evolve from a low-quality participation state to a medium-high quality state, while weakening policy incentives may lead the system to deteriorate to a low-quality state. This relationship reveals the dynamic correlations and conditional dependencies behind the multi-equilibrium pattern. $E_{\text{1}\text{6}}$ represents a situation where all four parties adopt proactive strategies, which is theoretically the most ideal state. However, it does not possess evolutionary stability in the replicator dynamics system. The fundamental reason is that when local governments, enterprises, and certification institutions have all actively participated and achieved high-quality engagement, consumers’ additional attention cannot further improve environmental performance. Instead, it incurs an attention cost, resulting in marginal benefits being less than marginal costs, thereby generating a motivation to abandon attention. Under the replicator dynamics of evolutionary games, some consumers’ shift from intense to mild attention gradually reduces the value, causing the state to converge from $E_{\text{1}\text{6}}$ to $E_{\text{1}\text{5}}$. Therefore, $E_{\text{1}\text{6}}$ lacks stability conditions and will not be spontaneously maintained in the long-term evolution.

## 3 Evolutionary path simulation and sensitivity analysis

As voluntary environmental regulations involve multiple subjects such as local governments, enterprises, certification institutions and consumers, their interaction is complex. It is not only highly impractical to comprehensively collect all the necessary data in a single case, but also difficult to achieve in public statistical data, and some information is even hard to obtain. Therefore, this study mainly relies on the research results of predecessors for parameter estimation. The assignment methods in relevant literature by Chen [7], Zhou [22], Zhao [23], Su [24], Jia [25] and others are referred to for reasonable and autonomous assignment of parameters in the model. Numerical simulation is carried out with MATLAB R2023(a), so as to the impact effects and action mode of high-quality participation in voluntary environmental regulation can be intuitively presented.

### 3.1 Evolutionary path simulation

**Fig 3:**
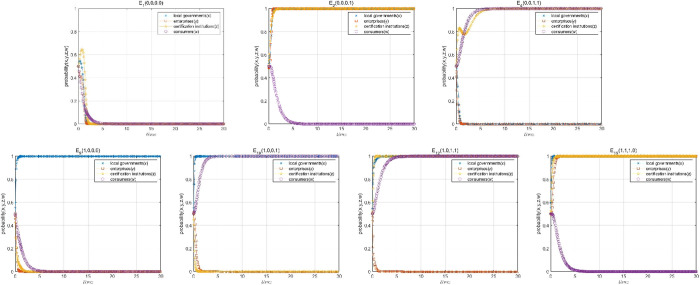
Evolutionary path at equilibrium point ${𝐄}_{1},\;{E_{2},E_{4},E}_{9},\;E_{10},\;E_{12},\;E_{15}$.

Setting the initial time as 0 and the end time as 30, the two-dimensional evolution paths of the four-party environmental behavioral strategies corresponding to the seven conditional stable equilibrium points of the voluntary environmental regulation high-quality participation game model are plotted with the initial probability [0.5,0.5,0.5,0.5] respectively, so as to present the equilibrium strategy tendency of all parties under the initial probability, as shown in Fig 3. The strategic tendency of each game player has different degrees of fluctuations and oscillations in the early stage of evolution in the situation corresponding to the 7 equilibrium points.

On the basis of measuring relative benefits, local governments choose active promotion strategy to encourage the high-quality participation of enterprises in most cases, which is a positive factor to enhance the high-quality participation level of voluntary environmental regulation, but it is easy to cause enterprises that do not meet the application conditions to collude with illegal certification institutions and obtain subsidies. Comparing the two scenarios $E_{\text{4}}$ and $E_{\text{1}\text{2}}$, the active promotion of the local governments can speed up the evolution of the certification institutions to choose the legal certification, and form the environmental governance direction of local governments’ supervision on certification institutions and certification institutions’ review on enterprises.

Enterprises choose qualified application strategy or not mainly depend on whether the certification institutions are legal certification. When the local governments actively promote, enterprises choose qualified applications and unqualified applications with the same probability. At this time, only the certification institutions choose legal certification strategy, enterprises will present relatively stable qualified application strategies. Therefore, legal certification by certification institutions is a key factor affecting the high-quality participation level of voluntary environmental regulation, and its importance is higher than that of local government subsidies.

Consumer concern has the least impact on improving the high-quality participation level of voluntary environmental regulation, which is lower than the active promotion by local governments and legal certification by certification institutions. Despite the existence of central ecological and environmental protection inspectors, when local governments are negative and neglectful ($E_{\text{1}}$ and $E_{\text{2}}$), the level of high-quality participation in voluntary environmental regulation is still low, which means that the punishment from the central government on local governments for dereliction of duty will not promote local governments to choose active promotion strategy.

Consistent with the results of the theoretical analyses, the environmental governance scenarios when the high-quality participation level of voluntary environmental regulation is increased is described as ${E_{\text{4}}(\text{0},\text{0},\text{1},\text{1}),E_{\text{1}\text{2}}(\text{1},\text{0},\text{1},\text{1}),E}_{\text{1}\text{5}}(\text{1},\text{1},\text{1},\text{0})$. $E_{\text{1}}(\text{0},\text{0},\text{0},\text{0}),E_{\text{2}}(\text{0},\text{0},\text{0},\text{1}),E_{\text{9}}(\text{1},\text{0},\text{0},\text{0})$ and $E_{\text{1}\text{0}}(\text{1},\text{0},\text{0},\text{1})$ gradually become stable in the long-term evolution, but the corresponding situation is not ideal, which is the most undesirable state of governmental failure and market failure in reality, and the high-quality participation level of voluntary environmental regulation is extremely low.

### 3.2 Sensitivity analysis

In the four-party evolutionary game model presented in this paper, the purpose of the sensitivity analysis is to examine the impact of key parameters with different values on the system’s evolutionary trajectory and the stability of equilibrium points, thereby verifying the robustness of the conclusions and analyzing the effectiveness of policy adjustments. The selection of factors mainly follows the following criteria: (1) In the replicator dynamic equations, some variables directly appear in the payoff functions of multiple players and have significant decision-making driving forces. Changes in such parameters will substantially alter the fitness of strategy choices and the evolutionary path of the system. B1 directly affects the net benefits of enterprises meeting the conditions for application and also indirectly adjusts the strategy payoffs of local governments and certification institutions. It is a core policy tool for incentivizing high-quality participation by enterprises. F1 directly increases the cost of non-compliant certification and significantly alters the payoff comparison between compliance and non-compliance strategies for certification institutions. It also indirectly suppresses the non-compliant application intentions of enterprises. β reflects the additional penalties imposed by media supervision on violations in economic and reputational terms. It affects the compliance choices of certification institutions and increases the reputational risks of enterprises’ non-compliant behaviors. It is an important intensity variable in the external supervision system. J reflects the enterprises’ compensation. The trade-off between the costs consumers pay for choosing a strong concern strategy and the potential benefits they may obtain determines the dynamic changes in their strategies. Against the backdrop of growing environmental awareness, consumers are gradually playing a more active role. (2) B1, F1, J and β are all adjustable and designable key tools in real policy and regulation, which have direct significance for achieving the policy goals of voluntary environmental regulations. B1 can be adjusted through fiscal policies, green finance, etc.; F1 is an administrative penalty standard that can be directly formulated by local market regulatory departments and national certification regulatory agencies; β can be increased by improving information disclosure quality, encouraging media supervision, and establishing a blacklist system; J mainly relies on the proactive actions of local governments for adjustment, such as improving the effectiveness of rights protection through the establishment of sound policy tools and institutional guarantees. Selecting these variables for sensitivity analysis has obvious policy simulation value and helps provide predictions of the effects of actions under different policy intensities for decision-making departments. Therefore, F1, B1, J and β are selected for sensitivity analysis to effectively reflect the differences in the formation mechanism of high-quality participation under different intensities of policy tools.

In terms of parameter values, first, the real intervals in the policy practices of some provinces and cities in China regarding green production, environmental certification, and pollution control were referred to. For instance, the local governments’ subsidies B1: ChengDu offers a one-time reward of 10,000 yuan to enterprises that pass the Environmental Management System (ISO14001) certification; WuHu rewards 20,000 yuan; and SanYa rewards 30,000 yuan. Considering the differences in economic development levels, fiscal capabilities, and policy goals among different regions, these actual amounts were moderately standardized in the model and mapped to 5 (low level), 10 (medium level), and 15 (high level) in the simulation, thus having policy representativeness. Secondly, setting different levels of incentive parameters in the evolutionary game simulation conforms to the basic idea of sensitivity analysis, facilitating the exploration of the dynamic evolution paths and stable states of participation strategies under different subsidy intensities. Finally, this setting also references the treatment methods in related existing studies to ensure the theoretical comparability and practical interpretability of the simulation process. Therefore, the selected subsidy levels strike a balance between theoretical rationality and policy representativeness.

#### 3.2.1 Sensitivity analysis of the subsidies to certified enterprises B1.

**Fig 4:**
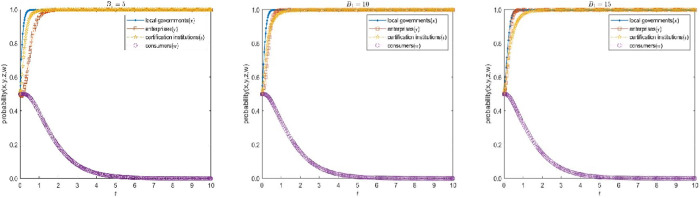
Influence analysis of the subsidies 𝐁1 on the evolution speed of enterprises’ choice of qualified application strategy.

Now the Subsidies to certified enterprises B1 of $E_{\text{1}\text{5}}$(1,1,1,0) in the high-quality participation model is assigned as 5, 10 and 15, the values of other parameters remain unchanged, and assigned as $\begin{array}{c} G\text{1}=\text{3}\text{0},\;C_{\text{1}}=\text{5},\;C_{\text{2}}=\text{1}\text{2},\;C_{\text{2}}^{\prime }=\text{6},\;C_{\text{3}}=\text{3},\;C_{\text{3}}^{\prime }=\text{1},\;C\text{4}=\text{1},\;\alpha =\text{1}.\text{1},\;S\text{1}=\text{1}, \end{array}$$\begin{array}{c} \beta =\text{1}.\text{1},\;S\text{2}=\text{4},\;R\text{1}=\text{4},\;H\text{1}=\text{4},\;H\text{2}=\text{6},\;F\text{1}=\text{5},\;F\text{2}=\text{1}\text{0},\;D\text{1}=\text{1},\;D\text{2}=\text{5},\;f=\text{5},\;J=\text{2},\;T=\text{2} \end{array}$ respectively. Under the same initial probability, time and step size, a comparative simulation is conducted to analyze how the subsidy factor of local government affects the evolution speed of the enterprise’s application strategy. The simulation results are shown in Fig 4.

As can be seen from Fig 4, there is negative correlation between local government’s choice of active promotion strategy and its subsidy, and positive correlation between local government’s subsidy and enterprises’ choice of qualified application strategy. When the subsidies of local governments B1 are small, local governments choose to active promotion voluntary environmental regulations with a faster evolution speed, local governments are less constrained by funds, so they are more likely to choose the active promotion strategy, thus contributing to the improvement of local environmental quality. On the contrary, when the subsidies of local governments B1 are large, local governments choose to active promotion voluntary environmental regulations at a slow pace, at this time, the subsidies of local governments to some extent make up for emission reduction costs and certification costs, the evolution speed of enterprises choosing qualified application strategy is faster.

It is worth noting that although the increase of subsidies by local governments can accelerate the evolution of enterprises choosing qualified applications strategy, the evolution speed of local governments choosing the active promotion strategy will slow down with the increase of subsidies, which will curb the enthusiasm of local governments to implement voluntary environmental regulation. Therefore, local governments need to keep the subsidies within a reasonable range in order to enhance the enthusiasm of enterprises and improve the high-quality participation level of voluntary environmental regulation.

#### 3.2.2 Sensitivity analysis of the fines to illegal certification institutions F1.

**Fig 5:**
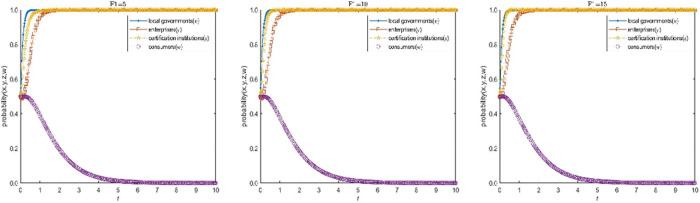
Influence analysis of the fines 𝐅1 on the evolution speed of certification institutions’ choice of legal certification strategy.

Now the fines to illegal certification institutions F1 when enterprises meet the certification conditions of $E_{\text{1}\text{5}}(\text{1},\text{1},\text{1},\text{0})$ in the high-quality participation model is assigned as 5, 10 and 15, the values of other parameters remain unchanged, and assigned as $G\text{1}=\text{3}\text{0},\;C_{\text{1}}=\text{5},\;C_{\text{2}}=\text{1}\text{2},\;C_{\text{2}}^{\prime }=\text{6},\;C_{\text{3}}=\text{3},\;C_{\text{3}}^{\prime }=\text{1},\;C\text{4}=\text{1},\;\alpha =\text{1}.\text{1},\;S\text{1}=\text{1},\;\beta =\text{1}.\text{1},\;S\text{2}=\text{4},\;R\text{1}=\text{4},\;H\text{1}=\text{4},\;H\text{2}=\text{6},\;B\text{1}=\text{5},\;F\text{2}=\text{1}\text{0},\;D\text{1}=\text{1},\;D\text{2}=\text{5},\;f=\text{5},\;J=\text{2},\;T=\text{2}$ respectively. Under the same initial probability, time and step size, a comparative simulation is conducted to analyze how the fines to the illegal certification institution affect the evolution speed of the choice of legal certification strategy. The simulation results are shown in Fig 5.

As can be seen from Fig 5, the fines to illegal certification institutions by local governments is positively correlated with the choice of legal certification strategy by certification institutions, and with the choice of qualified application strategy by enterprises. When the fines to illegal certification institutions are low, the evolution speed of choosing legal certification strategies by certification institutions is slow, certification institutions reduce certification costs or charge higher certification fees through illegal certification to maximize their own economic benefits, and enterprises are more likely to choose unqualified applications strategies, which is not conducive to the improvement of local environmental quality. When the fines to illegal certification institutions are high, the evolution speed of choosing legal certification strategies by certification institutions is fast, certification institutions will take the initiative to choose legal certifications, assume environmental responsibility, and ensure the effectiveness of certification. Therefore, local governments need to increase the severity of punishment and increase the amount of punishment, to minimize the possibility of illegal certification to ensure the authenticity of enterprise certification, the accuracy of local government subsidies and the effectiveness of certification institutions.

#### 3.2.3 Sensitivity analysis of the loss amplification factor β.

**Fig 6:**
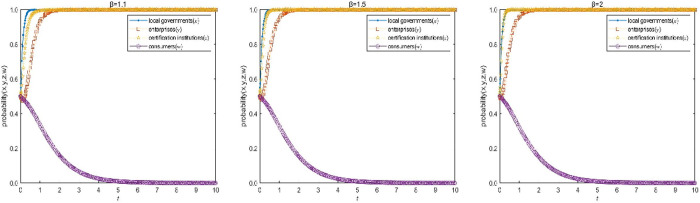
Influence analysis of the loss amplification factor β on the evolution speed of certification institutions’ choice of legal certification strategy.

The loss amplification factor β of the certification institutions under media exposure of $E_{\text{1}\text{5}}(\text{1},\text{1},\text{1},\text{0})$ in the high-quality participation model is assigned as 1.1, 1.5 and 2, the values of other parameters remain unchanged, and assigned as $G\text{1}=\text{3}\text{0},\;C_{\text{1}}=\text{5},\;C_{\text{2}}=\text{1}\text{2},\;C_{\text{2}}^{\prime }=\text{6},\;C_{\text{3}}=\text{3},\;C_{\text{3}}^{\prime }=\text{1},\;C\text{4}=\text{1},\;\alpha =\text{1}.\text{1},\;S\text{1}=\text{1},\;B\text{1}=\text{5},\;S\text{2}=\text{4},\;R\text{1}=\text{4},\;H\text{1}=\text{4},\;H\text{2}=\text{6},\;F\text{1}=\text{5},\;F\text{2}=\text{1}\text{0},\;D\text{1}=\text{1},\;D\text{2}=\text{5},\;f=\text{5},\;J=\text{2},\;T=\text{2}\;$respectively. Under the same initial probability, time and step size, a comparative simulation is conducted to analyze how the loss amplification factor β affect the evolution speed of the choice of legal certification strategy. The simulation results are shown in Fig 6.

As can be seen from Fig 6, the loss amplification factor β of the certification institutions under media exposure is positively correlated with the choice of legal certification strategy by certification institutions. When the amplification factor is low, the evolution speed of choosing legal certification strategies by certification institutions is slow, certification institutions are willing to bear a certain loss to maximize their own interests. When the amplification factor is high, the evolution speed of choosing legal certification strategies by certification institutions is fast, the increase of losses makes it difficult for certification institutions to firmly choose the illegal certification strategy under the cost-benefit measurement. At this time, the legal certification institution will strictly supervise and review the certification process of enterprises, and urge enterprises to reduce pollution and emission from the source. Therefore, local governments need to guide the media to increase the level and broaden the scope of exposure to minimize the possibility of illegal certification.

#### 3.2.4 Sensitivity analysis of the enterprises’ compensation to consumers J.

**Fig 7:**
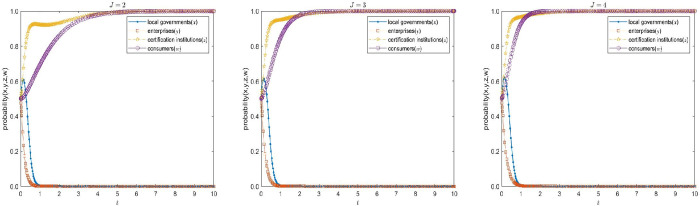
Influence analysis of the enterprises’ compensation to consumers on the evolution speed of consumers’ choice of strong concern strategy.

The enterprises’ compensation to consumers J of ${\text{E}}_{\text{4}}(\text{0},\text{0},\text{1},\text{1})$ in the high-quality participation model is assigned as 2, 3 and 4, the values of other parameters remain unchanged, and assigned as $G\text{1}=\text{3}\text{0},C_{\text{1}}=\text{1}\text{0},C_{\text{2}}=\text{2}\text{0},C_{\text{2}}^{\prime }=\text{6},C_{\text{3}}=\text{3},C_{\text{3}}^{\prime }=\text{1},C\text{4}=\text{1},\alpha =\text{1}.\text{1},\beta =\text{1}.\text{1},S\text{1}=\text{1},B\text{1}=\text{5},S\text{2}=\text{4},R\text{1}=\text{4},H\text{1}=\text{5},H\text{2}=\text{6},F\text{1}=\text{5},F\text{2}=\text{1}\text{0},D\text{1}=\text{1},D\text{2}=\text{1},f=\text{5},T=\text{2}\;$respectively. Under the same initial probability, time and step size, a comparative simulation is conducted to analyze how the enterprises’ compensation to consumers J affect the evolution speed of the choice of strong concern strategy. The simulation results are shown in Fig 7.

As shown in Fig 7, the enterprises’ compensation to consumers is positively correlated with the consumers’ choice of the strong concern strategy. When the enterprises’ compensation to consumers is low, their willingness to participate is insufficient, and the evolution speed of the strong concern strategy is relatively slow; while when the enterprises’ compensation to consumers is high, consumers are more inclined to choose the strong concern strategy, thereby significantly accelerating the evolution process of this strategy. However, even in the context of consumers’ strong concern, enterprises can still obtain short-term benefits through non-compliant certification, demonstrating the continuous existence of opportunistic behavior. This indicates that consumers cannot completely restrain enterprises’ non-compliant behavior. Therefore, to achieve high-quality participation in voluntary environmental regulations, it is not only necessary to encourage consumers to actively protect their rights, but also requires local governments to guide enterprises to internalize environmental compliance as a long-term competitive advantage by improving policy incentives and punishment mechanisms.

## 4 Conclusions and policy recommendations

### 4.1 Conclusions

There are nine unstable equilibrium points and seven conditionally stable equilibrium points in the four-way evolutionary game model under the framework of voluntary environmental regulation, corresponding to the real situation, the ideal degree of each conditional stable point is quite different, which can be seen by comparison:

(Ⅰ) Active promotion by local governments, legal certification by certification institutions, and strong concern by consumers can all increase the level of high-quality participation in voluntary environmental regulation. Among them, legal certification is a key factor to improve the high-quality participation level of voluntary environmental regulations, which is more important than the active promotion and consumers’ strong concern; consumers’ strong concern has the weakest effect.
(Ⅱ) The environmental governance direction of “local governments supervise certification institutions - certification institutions review enterprises” has been confirmed, and certification institutions play a key role in improving the level of high-quality participation in voluntary environmental regulation. When local governments actively promote and strictly supervise certification institutions, certification institutions will accelerate the evolution of legal certification strategies. In the process of legal certification, the certification institutions will strictly supervise and review the qualification of the enterprises in a professional and rigorous attitude to ensure the effectiveness and authority of the certification.
(Ⅲ) Increased subsidies by local governments can help enterprises choose qualified application strategies more quickly, and increased penalties by local governments and increased media exposure can help certification institutions choose legal certification strategies more quickly. Some enterprises do not actively participate in voluntary environmental regulations because they are difficult to bear the cost of green production and certification fee. the subsidies from local governments can reduce the economic pressure of enterprises and increase their enthusiasm for participation. In addition, the excessive pursuit of profit maximization by certification institutions in the process of providing certification services may lead to the tendency of illegal operations. The strict supervision by local governments and the in-depth exposure of illegal behaviors by the media will cause direct economic losses and reputational damage to certification institutions and reduce the possibility of illegal certification.
(Ⅳ) The penalties imposed by the central government on local governments for dereliction of duty do not significantly affect the strategic choices of local governments. The reason for this may lie in the highly international and authoritative nature of the ISO14001 standard, which provides unified standards and guidance for environmental protection and sustainable development on a global scale. The main responsibility of the central government is to promulgate and promote this standard to ensure that it is in line with international standards and adapt to the actual situation in China, but the real high-quality implementation of the standard depends more on the efforts of local governments.

### 4.2 Policy recommendations

Based on the above conclusion, to promote high-quality participation in voluntary environmental regulations, policy formulation should simultaneously exert efforts in three dimensions: precise incentives, strict supervision, and strengthened public opinion supervision, establishing a closed loop from incentives to constraints.

Firstly, in terms of subsidy and financial support policies, it is suggested that the local finance bureau take the lead and the market supervision and administration bureau cooperate to establish a subsidy distribution mechanism of “process evaluation + result assessment”: before enterprises obtain certification, set necessary regulatory nodes for the review process (such as document review, on-site inspection records, proof of rectification completion), and conduct random checks after certification to ensure that fiscal subsidies flow to truly compliant enterprises. At the same time, the simulation results show that a moderate level of fiscal subsidies can significantly increase the speed at which enterprises meet the conditions for application without dampening the enthusiasm of local governments. Therefore, in budget allocation, local governments should control the subsidy amount within the optimal range between incentive effect and fiscal sustainability to avoid the weakening of government promotion due to “excessive subsidies”; and can further reduce the long-term compliance costs of enterprises by relying on green financial tools (such as policy-based low-interest loans, tax and fee reductions, green credit).

Secondly, in terms of the supervision and penalty mechanism for certification institutions, it is suggested that the National Certification and Accreditation Administration and local market supervision and administration bureaus work in tandem to establish a tiered penalty system and enforce it strictly. For minor violations (such as lax review in certain stages), medium-sized fines should be imposed and rectification within a specified period should be required. However, for serious violations (such as issuing certifications for non-compliant enterprises), measures such as revoking qualifications, listing on the national “blacklist”, and imposing heavy fines should be taken to ensure that the cost of violations is significantly higher than the gains from violations. Sensitivity analysis shows that raising the level of penalties can significantly accelerate the evolution speed of certification institutions towards compliance, and fundamentally reduce the probability of non-compliant certifications. In addition, an annual credit evaluation system should be established for certification institutions, linking compliance rates, violation records with the qualification to undertake certification business, and giving priority recommendations and government procurement preferences to high-scoring institutions.

Finally, in terms of media supervision and information disclosure mechanisms, the Publicity Department of the Municipal Committee and the Cyberspace Administration should take the lead in building provincial and municipal environmental protection certification public opinion monitoring and information disclosure platforms, connecting with the law enforcement data of the State Administration for Market Regulation and local ecological environment bureaus, to form a closed-loop mechanism of identifying violations – rapid verification – authoritative release – continuous tracking. For major violations, mainstream media should be required to report publicly within 24 hours, and for general violations, a regular “rolling exposure” approach should be adopted to continuously exert pressure. At the same time, an “exposure archive” of violating enterprises and institutions should be launched on the platform for consumers and partners to query at any time. Simulation results show that increasing the amplification coefficient of media exposure losses can significantly enhance the reputation and economic loss multiplier effect of violations, thereby forcing certification institutions to comply. On this basis, local governments can establish an “environmental protection supervision reward fund” to provide cash rewards or points redemption to individuals or organizations that provide effective violation clues and successfully promote the filing and investigation, in order to stimulate the enthusiasm of the public and social organizations to participate in environmental governance.
